# Orofacial features and medical profile of eight individuals with Kabuki syndrome

**DOI:** 10.4317/medoral.22982

**Published:** 2019-08-19

**Authors:** Natália Silva-Andrade, Karem López-Ortega, Marina Gallottini

**Affiliations:** 1 PhD Student of University of São Paulo, São Paulo, Brazil; 2 Full Professor of University of São Paulo, São Paulo, Brazil

## Abstract

**Background:**

To evaluate oral, craniofacial and systemic characteristics of eight patients with Kabuki syndrome (KS), aged between 3 and 16 years old.

**Material and Methods:**

In this retrospective study, medical records of all patients were reviewed for information on family history, growth and development, medications in use, general systemic complications and oral and craniofacial characteristics.

**Results:**

The medical alterations found included recurrent infections such as pneumonia and otitis media (n = 6), cardiovascular malformations (n = 4), kidney abnormalities (n = 2), epilepsy (n = 2) and visual deficiency (n = 2). The individuals exhibited dental caries (n = 5), agenesis (n = 5), delayed tooth eruption (n = 4), cleft lip/palate (n = 2) enamel hypoplasia (n = 2), fusion (n = 1) and microdontia (n = 1).

**Conclusions:**

There was a great diversity of oral, craniofacial and systemic characteristic among the KS patients, suggesting that an inter-disciplinary approach should be taken for their dental treatment.

** Key words:**Kabuki syndrome, oral manifestations, medical alterations.

## Introduction

Kabuki syndrome (KS, OMIM 3147920, 3300867), also known as Kabuki make-up syndrome or Niikawa-Kuroki syndrome, is a rare genetic disorder characterised by multiple congenital anomalies and intellectual disability (1,2). This syndrome was first described in Japan in 1981 by Niikawa & Kuroki. They reported, in two independent studies in the same year, the characteristics of 42 individuals presenting peculiar facial features, skeletal and dermatoglyphic abnormalities, postnatal growth deficiency and mild to moderate intellectual disability. Its name was suggested because the facial dysmorphism present in this condition resembled the Kabuki masks used by actors in the traditional Japanese theatre (3,4).

The majority of the cases reported in the literature are sporadic, but the presence of KS individuals within the same family has suggested an autosomal dominant inheritance. Although chromosomal anomalies have been associated with this syndrome (5), mutations in genes KMT2D/MLL2 and KDM6A are considered, nowadays, the main causes of KS (6-8).

Until recently, this condition was under-diagnosed in other populations other than the Japanese, with incidence ranging from 1:32,000 to 1:86,000 and no gender, ethnic or age prevalence.2 The diagnosis of KS is clinical and based on five major characteristics (1,9) as follows.

1. Facial dysmorphism: long palpebral fissure with eversion of the lateral third of the lower eyelids, arched eyebrows with hair rarefaction on the lateral third, long curved eyelashes, hypertelorism, large prominent earlobes, wide nose with depressed nasal tip;

2. Skeletal abnormalities: brachydactyly of fingers and toes, clinodactyly, deformed vertebrae or ribs, dislocation of the hip joints and patella;

3. Dermatoglyphic abnormalities: presence of digital pads and inner loops, absence of the digital c and/or triradii, increased digital ulnar and hypothenar loops patterns;

4. Mild-to-moderate intellectual disability;

5. Postnatal growth deficiency.

In addition to these characteristics, patients diagnosed with KS may also present cardiovascular, respiratory, renal, hepatic and gastrointestinal impairments, including presence of neurological alterations, susceptibility to infections and visual and hearing deficiencies (10-12).

Oral manifestations are present in more than 60 percent of the KS individuals,5 with high palate, cleft lip/palate, bifid tongue and uvula, malocclusion (micrognathia, retrognathia, diastema), tooth agenesis and delayed tooth eruption being the most frequent ones. Other changes, less frequently reported, include fusion, gemination, microdontia, taurodontism, external root resorption, enamel hypoplasia and ectopic tooth eruption (5,13-17).

The anatomical and functional complexity, in association with behavioural changes of the KS individuals, poses a challenge to dentists as they have to adjust the dental clinical management in order to prevent complications and to implement a treatment planning accordingly. Therefore, the objective of the present study was to evaluate the oral, craniofacial and systemic characteristics of patients diagnosed with Kabuki syndrome.

## Material and Methods

The research ethics committee of the Faculty of Dentistry of the University of São Paulo (CEP/FOUSP) approved this retrospective study (Protocol Nº 2.635.622), and it met the ethics recommendations dictated by the Declaration of Helsinki.

The present study describes all the male and female patients of different ages who were genetically diagnosed with KS and had been attending the Centre for Special Patients of the University of São Paulo Faculty of Dentistry (CAPE-FOUSP). Medical records of all patients were reviewed for information on family history (i.e. pre-natal, peri-natal and post-natal data such as mother’s gestational age, occurrences of abortion and infectious diseases during pregnancy, birth term, presence of peri-natal cyanosis), growth and development (i.e. age of speech and delay in postnatal growth), medications in use and general systemic complications (i.e. cardiac defects, gastrointestinal, renal and hepatic disorders, visual and hearing anomalies, among others).

Oral and craniofacial characteristics have been evaluated by means of clinical examination performed by calibrated examiners (i.e. the own authors), who used probe and mouth mirror under direct light. Oral and dental abnormalities were evaluated as follows: presence of caries, presence of absence of cleft lip/palate, presence or absence of developmental dental abnormalities, presence of malocclusions, high-arched palate and typical facial characteristics of KS.

## Results

Eight patients diagnosed with KS were evaluated, being three girls and five boys aged between 3 and 16 years old and no consanguineous. Three of them exhibited mutation in MLL2 gene. Although there was no family history of KS. Two cases reported occurrence of Down’s syndrome and Rett’s syndrome affecting relatives. Two patients were born prematurely, in two other pregnancies there was abortion threat and four patients presented peri-natal cyanosis, with all participants presenting delay in their speech development ( Table 1).

**Table 1:**
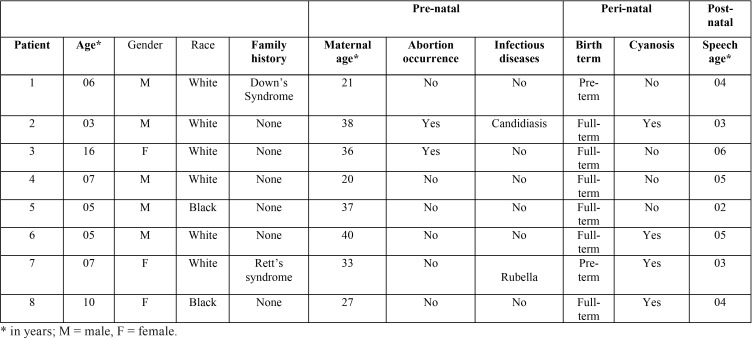
Demographic and medical history data of the eight patients with Kabuki syndrome during perinatal period.

Data on diagnostic characteristics and systemic manifestations of the eight patients affected by KS are briefly listed in  Table 2,  Table 2 continue. The majority of the patients (n = 5) exhibited five of the major characteristics related to KS, with the most common systemic manifestations being recurrent infections (i.e. pneumonia, otitis, rhinitis, sinusitis and urinary infection), cardiovascular malformations, congenital renal anomalies and seizures.

**Table 2:**
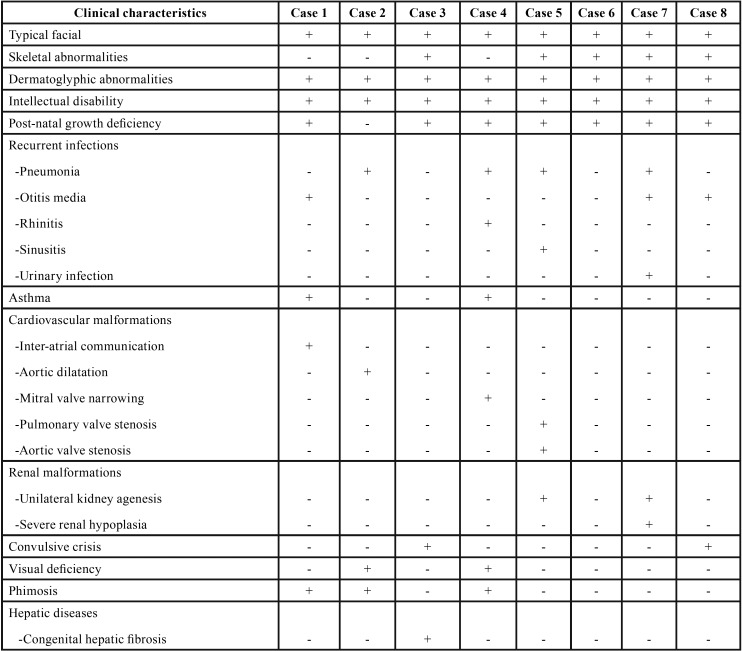
Clinical characteristics and medical history of the eight patients with Kabuki syndrome.

**Table 2 continue:**
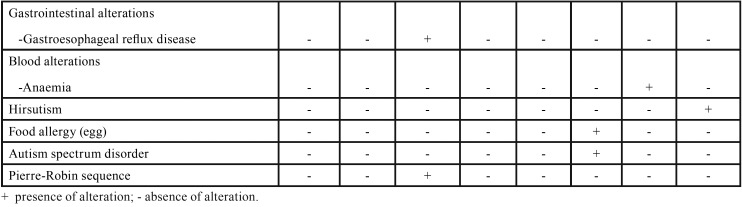
Clinical characteristics and medical history of the eight patients with Kabuki syndrome.

**Figure 1:**
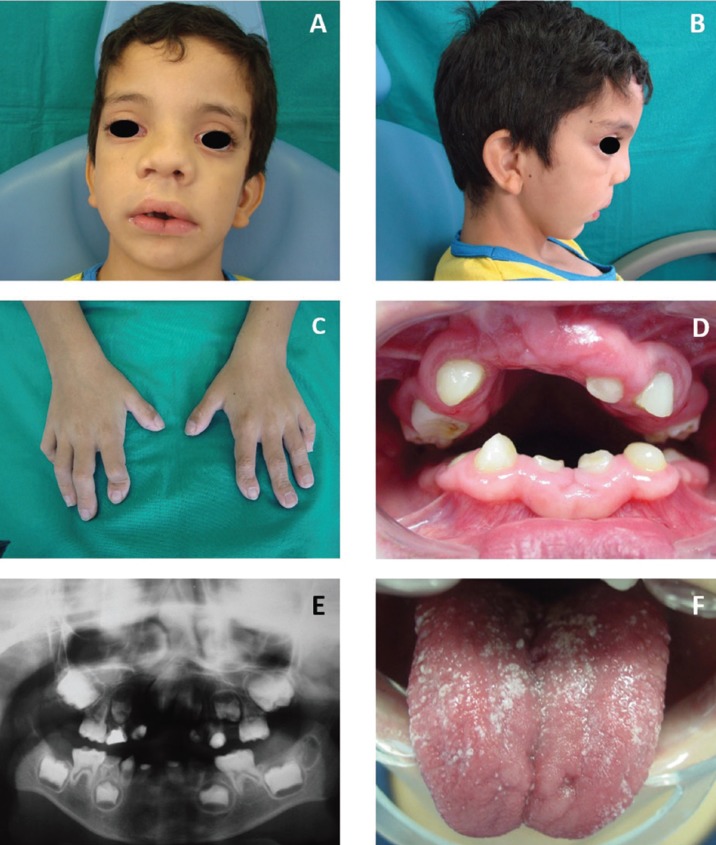
Patient #5. A) Frontal view of the face showing long palpebral fissure with eversion of the lateral third of the lower eyelids, hypertelorism, strabismus, sparse eyebrows in the lateral third, wide nose and surgical scar of a cleft lip-palate correction. B) Lateral view of the face showing protruding ears with low implantation and concave profile. C) View of the patient’s hands showing brachydactyly of the fifth finger. D) Intra-oral view showing spaced teeth, absence of teeth and enamel hypoplasia. E) Panoramic radiograph showing dental agenesis. F) Bifid tongue with pseudomembranous candidiasis.

**Figure 2:**
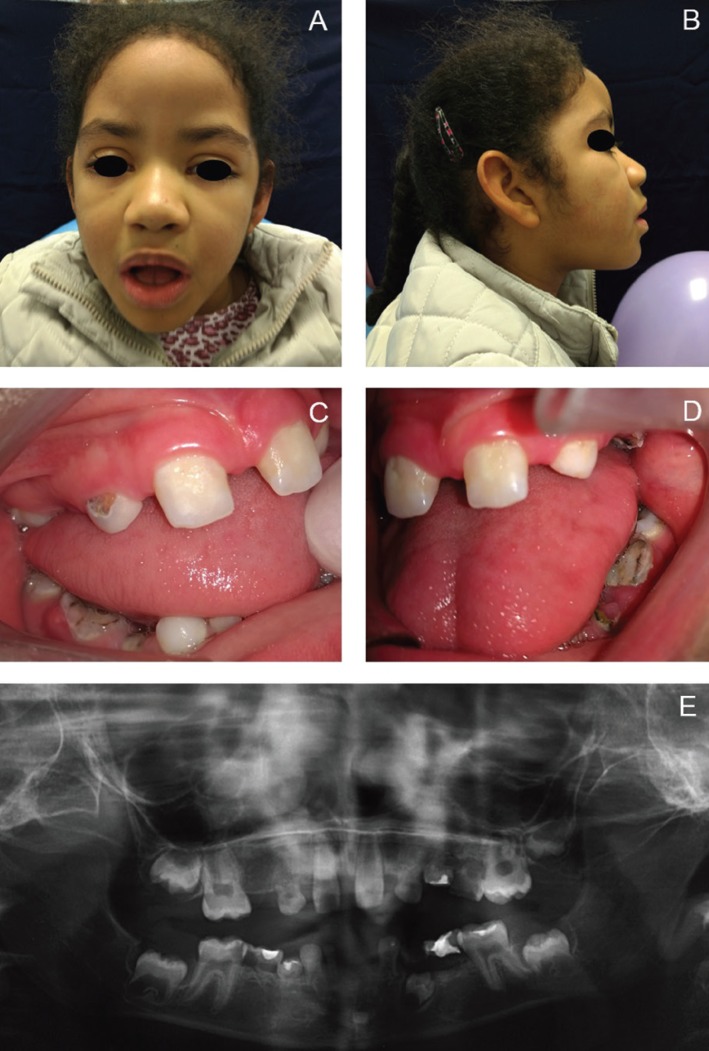
Patient #8. A) Frontal view of the hypotonic face showing long palpebral fissure with eversion of the lateral third of the lower eyelids, hypertelorism, palpebral ptosis, arched and sparse eyebrows in the lateral third, and wide nose. B) Lateral view of the face showing protruding ears with low implantation and depressed nasal bridge. C) Intra-oral right view showing spaced teeth, absence of teeth, enamel hypoplasia and caries lesions. D) Intra-oral left view showing spaced teeth, absence of teeth, enamel hypoplasia and caries lesions. D) Panoramic radiograph showing dental agenesis and prolonged retention of tooth #41.

The facial characteristics of the patients examined included long palpebral fissure with eversion of the lower lateral third eyelids, high-arched eyebrows with a sparse or dispersed on the lateral side, large prominent earlobes and wide nose with depressed nasal tip and bridge (Figs. 1,2).

All the individuals presented with some alteration in the oral cavity. Six had high palate, five had tooth agenesis and two had cleft lip/palate. Active carious lesions were observed in five patients ( Table 3).

**Table 3:**
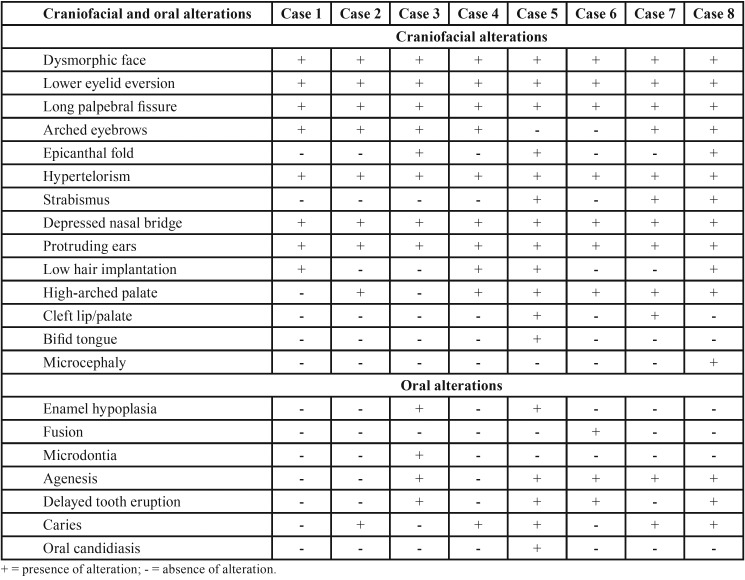
Craniofacial and oral alterations in the eight patients with Kabuki syndrome.

## Discussion

The diagnosis of Kabuki syndrome is clinically determined on the basis of five major characteristics: peculiar facial features, skeletal abnormalities, dermatoglyphic alterations, intellectual disability and post-natal growth deficiency (1,9). The two genes related to the KS etiology (i.e. KMT2D/MLL2 and KDM6A) work as epigenetic modulators in several biological processes through modifications of histones throughout the embryogenesis.6-8 This would be the reason of great heterogeneity of manifestations presented in KS patients, such as multiple congenital malformations, developmental defects, disturbed growth and also haematological and immunological defects (12) 

Renal malformations were present in 22 percent of the cases in a French cohort study involving 94 KS individuals, with 28 percent of the MLL2-mutation positive group presenting renal malformations and no case in the MLL2-mutation negative group (*P* = 0.015). In the same study, the authors identified a patient with unilateral kidney agenesis and severe contra-lateral hypoplasia (18). In our report, two patients presented unilateral renal agenesis and one of them had severe malformation of the remaining kidney. The implication of kidney disease in the dental management of these patients is mainly related to a judicious prescription of nephrotoxic drugs and increased risk of bleeding (19).

Hepatic diseases, such as congenital liver fibrosis, have already been reported in individuals with KS (20) and in our study only one participant was diagnosed with chronic hepatopathy. Simple or complex congenital cardiac defects have been diagnosed in 30-55 percent of the KS individuals, with the most common abnormalities being vascular obstructions/dilatations and septal defects, including aortic coarctaction, aortic stenosis, atrial and ventricular septal defects, bicuspid aorta, tetralogy of Fallot, among others (21). The presence of congenital cardiopathies in these patients increases the risk of occurrence of infectious bacterial endocarditis, and according to the most recent recommendations of the American Heart Association, the use of antibiotic prophylaxis prior to invasive dental procedures is recommended for these individuals (22). In the present study, four individuals had cardiovascular malformations (i.e. Inter-atrial communication, stenosis of pulmonary and aortic valves, aorta dilatation and mitral valve narrowing).

Pneumonia, otitis, sinusitis and urinary infection were the recurrent infectious diseases reported in our study. Due to the decrease in the levels of immunoglobulins (e.g. IgA, IgG and IgM) and memory cells (CD19+ and CD4+), the KS patients are more susceptible to these infections (10,12,23). Despite that, only one participant (#5) presented oral pseudomembranous candidiasis as confirmed by cytological examination, who responded favourably to treatment with nystatin mouth rinse for 15 days.

The intellectual disability and delayed speech seen in all our patients contribute to the impairment in their language development by altering the expressive and receptive skills as well as by reducing the grammar ability (24), which makes communication and dental care difficult. In addition to the intellectual disability, the motor disability resulting from skeletal changes affects the oral hygiene of these patients and increases the incidence of caries, which was present in five of the eight patients included in this study (16,25).

The oral abnormalities most frequently observed in the eight patients were high palate, tooth agenesis and cleft lip/palate. Dental developmental disorders mostly reported in the literature include microdontia, microdontia, external root resorption, fusion, gemination, ectopic eruption, delayed tooth eruption, enamel hypoplasia, cone- and screwdriver-shaped incisors (1,14,16,17,25,26) In a case report of a 9-year-old boy, Rocha *et al.* (15) described supernumerary teeth and taurodontism, which are frequently found in KS patients. Hypodontia and diastemas in KS patients can result in occlusal alterations, leading to the need for orthodontic treatment (16,17,25). In the patients described in this study, the lower second premolars were the teeth mostly commonly affected by agenesis, followed by lateral incisors and lower canines. One patient had agenesis of the upper right canine, thus confirming findings already reported in the literature.16 

All the patients in our study were treated in an outpatient clinic, and despite their intellectual disability, we had cooperation from all for their dental treatment. Dentists should be aware of the presence of chronic systemic diseases such as cardiopathies, nephropathies and hepatopathies. Their dental care should include antibiotic prophylaxis for those at high risk of bacterial endocarditis in situations involving invasive dental procedures. For those presenting renal failure, care was taken to avoid prescribing any nephrotoxic drug, especially non-steroidal anti-inflammatory ones. Patients with history of asthma always had at hand bronchodilator drugs during the dental treatment. Because the majority of the participants had caries lesions, both patients and their caregivers were strongly instructed to maintain a good oral health.

## References

[1] Tuna EB, Marşan G, Gençay K, Seymen F (2012). Craniofacial and dental characteristics of Kabuki syndrome: nine years cephalometric follow-up. J Clin Pediatr Dentist.

[2] Adam MP, Hudgins L (2005). Kabuki syndrome: A review. Clin Genet.

[3] Niikawa N, Matsuura N, Fukushima Y, Ohsawa T, Kajii T (1981). Kabuki make-up syndrome: a syndrome of mental retardation, unusual facies, large and protruding ears, and postnatal growth deficiency. J Pediatr.

[4] Kuroki Y, Suzuki Y, Chyo H, Hata A, Matsui I (1981). A new malformation syndrome of long palpebral fissures, large ears, depressed nasal tip, and skeletal anomalies associated with postnatal dwarfism and mental retardation. J Pediatr.

[5] Atar M, Lee W, O'Donnell D (2006). Kabuki syndrome: Oral and general features seen in a 2-year-old Chinese boy. Int J Paediatr Dent.

[6] Ng SB, Bigham AW, Buckingham KJ, Hannibal MC, McMillin M, Gildersleeve H (2010). Exome sequencing identifies MLL2 mutations as a cause of Kabuki syndrome. Nat Genet.

[7] Miyake N, Koshimizu E, Okamoto N, Mizuno S, Ogata T, Nagai T (2013;161A). MLL2 and KDM6A mutations in patients with Kabuki syndrome. Am J Med Genet.

[8] Dentici ML, Di Pede A, Lepri FR, Gnazzo M, Lombardi MH, Auriti C (2015). Kabuki syndrome: clinical and molecular diagnosis in the first year of life. Arch Dis Child.

[9] Matsumoto N, Niikawa N (2003). Kabuki make-up syndrome: a review. Am J Med Genet C Semin Med Genet.

[10] Geneviève D, Amiel J, Viot G, Le Merrer M, Sanlaville D, Urtizberea A (2004). Atypical findings in Kabuki syndrome: report of 8 patients in a series of 20 and review of the literature. Am J Med Genet.

[11] Liu S, Hong X, Shen C, Shi Q, Wang J, Xiong F (2015). Kabuki syndrome: a Chinese case series and systematic review of the spectrum of mutations. BMC Med Genet.

[12] Stagi S, Gulino AV, Lapi E, Rigante D (2016). Epigenetic control of the immune system: a lesson from Kabuki syndrome. Immunol Res.

[13] Matsune K, Shimizu T, Tohma T, Asada Y, Ohashi H, Maeda T (2001). Craniofacial and dental characteristics of Kabuki syndrome. Am J Med Genet.

[14] Dos Santos BM, Ribeiro RR, Stuani AS, Silva FWGDP, De Queiroz AM (2006). Kabuki make-up (Niikawa-Kuroki) syndrome: Dental and craniofacial findings in a Brazilian child. Braz Dent J.

[15] Rocha CT, Peixoto ITA, Fernandes PM, Torres CP, De Queiroz AM (2008). Dental findings in Kabuki make-up syndrome: A case report. Spec Care Dentist.

[16] Teixeira CS, Silva CRL, Honjo RS, Bertola DR, Albano LMJ, Kim CA (2009). Dental evaluation of Kabuki syndrome patients. Cleft Palate Craniofac J.

[17] Kumar H, Prabhu N, Cameron A (2009). KBG syndrome: review of the literature and findings of 5 affected patients. Oral Surg Oral Med Oral Pathol Oral Radiol Endod.

[18] Courcet JB, Faivre L, Michot C, Burguet A, Perez-Martin S, Alix E (2013). Clinical and molecular spectrum of renal malformations in Kabuki syndrome. J Pediatr.

[19] Gupta M, Gupta M, Abhishek (2015). Oral conditions in renal disorders and treatment considerations - A review for pediatric dentist. Saudi Dent J.

[20] Nobili V, Marcellini M, Devito R, Capolino R, Viola L, Digilio MC (2004). Hepatic fibrosis in Kabuki syndrome. Am J Med Genet.

[21] Yuan SM (2013). Congenital heart defects in Kabuki syndrome. Cardiol J.

[22] Wilson W, Taubert KA, Gewitz M, Lockhart PB, Baddour LM, Levison M (2007). Prevention of infective endocarditis: guidelines from the American Heart Association: a guideline from the American Heart Association. Circulation.

[23] Lin JL, Lee WI, Huang JL, Chen PK, Chan KC, Lo LJ (2015). Immunologic assessment and KMT2D mutation detection in Kabuki syndrome. Clin Genet.

[24] Defloor T, van Borsel J, Schrander-Stumpel CT, Curfs LM (2005). Expressive language in children with Kabuki syndrome. Am J Med Genet.

[25] Petzold D, Kratzsch E, Opitz C, Tinschert S (2003). The Kabuki syndrome: four patients with oral abnormalities. Eur J Orthod.

[26] Cogulu D, Oncag O, Celen E, Ozkinay F (2008). Kabuki Syndrome with additional dental findings: a case report. J Dent Child.

